# Unexpected Heme Redox Potential Values Implicate an Uphill Step in Cytochrome *b*_6_*f*

**DOI:** 10.1021/acs.jpcb.2c05729

**Published:** 2022-11-18

**Authors:** Mateusz Szwalec, Łukasz Bujnowicz, Marcin Sarewicz, Artur Osyczka

**Affiliations:** Department of Molecular Biophysics, Faculty of Biochemistry, Biophysics and Biotechnology, Jagiellonian University, Gronostajowa 7, 30-387Kraków, Poland

## Abstract

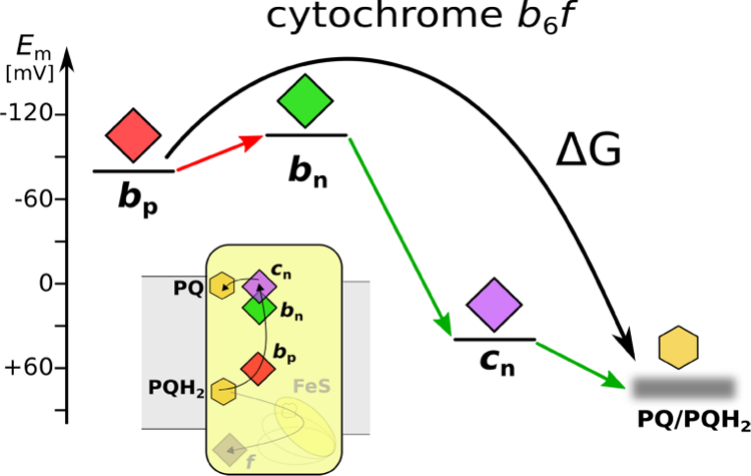

Cytochromes *bc*, key enzymes of respiration and photosynthesis, contain a highly conserved two-heme motif supporting cross-membrane electron transport (ET) that connects the two catalytic quinone-binding sites (Q_n_ and Q_p_). Typically, this ET occurs from the low- to high-potential heme *b*, but in photosynthetic cytochrome *b*_6_*f*, the redox midpoint potentials (*E*_m_s) of these hemes remain uncertain. Our systematic redox titration analysis based on three independent and comprehensive low-temperature spectroscopies (continuous wave and pulse electron paramagnetic resonance (EPR) and optical spectroscopies) allowed for unambiguous assignment of spectral components of hemes in cytochrome *b*_6_*f* and revealed that *E*_m_ of heme *b*_n_ is unexpectedly low. Consequently, the cross-membrane ET occurs from the high- to low-potential heme introducing an uphill step in the energy landscape for the catalytic reaction. This slows down the ET through a low-potential chain, which can influence the mechanisms of reactions taking place at both Q_p_ and Q_n_ sites and modulate the efficiency of cyclic and linear ET in photosynthesis.

## Introduction

Cytochrome *b*_6_*f* (cyt*b*_6_*f*) is a homodimeric, membrane-embedded enzyme playing a crucial role in oxygenic photosynthesis, in which it provides a functional connection between photosystems II and I.^1−3^ Cyt*b*_6_*f* is believed to operate according to the Q cycle mechanism originally formulated to describe the catalytic action of cytochrome *bc*_1_ (cyt*bc*_1_), a counterpart of cyt*b*_6_*f* involved in the molecular respiration in mitochondria and some bacteria. Both cyt*b*_6_*f* and cyt*bc*_1_ catalyze the electron transfer from membrane-soluble electron carriers plastoquinones (PQs) or ubiquinones (UQs), respectively, to water-soluble electron carriers plastocyanins or cytochromes (cyts) *c*, depending on the organism. The energy released during the electron transfer process is utilized to transport protons across the lipid membrane, thus contributing to the proton motive force required for ATP synthesis.^4−6^

The Q cycle is based on a joint operation of two catalytic sites: the quinone oxidation site (Q_p_ or Q_o_ in cyt*b*_6_*f* and cyt*bc*_1_, respectively) and the quinone reduction site (Q_n_ or Q_i_).^7−10^ The Q_p_ site catalyzes a bifurcation reaction, which directs electrons originating from the oxidation of quinol into two separate cofactor chains: the high-potential and the low-potential chain (LPC). One electron reduces the Rieske cluster and subsequently cytochrome *c* in the high-potential chain. The other electron reduces heme *b*_p_ (h*b*_p_) and is then transferred across the membrane to heme *b*_n_ (h*b*_n_) in the LPC. An electron from this heme is eventually used to reduce quinone to quinol at Q_n_ (because of the bifurcation, two reactions at Q_p_ yield electrons required for the reduction of one quinone at Q_n_). Thus, electron transfer from h*b*_p_ to h*b*_n_ links functionally the two catalytic sites.^1,4^

The two hemes *b* form a highly conserved structural motif. Accordingly, the spatial arrangement of these hemes is nearly identical in cytochromes *bc*_1_ and *b*_6_*f*.^11^ Given the direction of electron transfer, it is generally expected that h*b*_p_ possesses a lower redox midpoint potential (*E*_m_) than h*b*_n_.^12−14^ This indeed is the case for cytochrome *bc*_1_, for which the values of *E*_m_ of hemes *b* are well-established, being at pH 8.0 around −120 mV and +50/+150 mV (two components) for h*b*_p_ and h*b*_n_, respectively. For this reason, h*b*_p_ and h*b*_n_ are commonly denoted as h*b*_l_ and h*b*_h_, respectively (where “l” and “h” stand for low- and high midpoint potential, respectively). However, in the case of cyt*b*_6_*f*, the *E*_m_ values of respective hemes are much less clear, and a discussion on the redox properties of these hemes is ongoing.^15^ For long, the organization of the LPC of cyt*b*_6_*f* was considered to be highly similar to that of cyt*bc*_1_, but since the discovery of a *c*-type high-spin (HS) heme *c*_n_ (h*c*_n_) in cyt*b*_6_*f* located very close to h*b*_n_,^16,17^ the discussion now includes this additional component possibly forming an interacting redox pair with h*b*_n_.^18^

The major uncertainty concerns the actual difference in *E*_m_ between the h*b*_p_ and h*b*_n_.^15^ The analogy to cyt*bc*_1_ would imply a lower *E*_m_ for h*b*_p_ and a higher one for h*b*_n_, but there have been experimental indications that these hemes might in fact be isopotential. The experiments of Rich and Bendall^19^ suggested that midpoint potentials (*E*_m_s) of hemes *b* of cyt*b*_6_*f* isolated from higher plants are distinct, with reported *E*_m_s of +85 and −90 mV (pH 7.0). Accordingly, Hurt and Hauska observed well-separated *E*_m_s of hemes *b* with values of −60 and −188 mV at pH 7.4.^20^ Later, they re-evaluated these values by low-temperature optical measurements obtaining two partially overlapping spectral components, which yielded values of ∼3 mV for high-potential heme and −146 mV for low-potential heme at pH 5.6.^21^ Contrarily, the redox titrations of cyt*b*_6_*f* natively embedded in the photosynthetic membrane of spinach chloroplasts showed that hemes *b* are isopotential within 50 mV with *E*_m_ = −45 mV at pH 8.0.^22^ In yet another experiment, Clark and Hind^23^ titrated isolated cyt*b*_6_*f* at pH 7.5 and obtained *E*_m_s of −30 and −150 mV. Based on these values, Clark and Hind speculated that in order for cyt*b*_6_*f* to obey the Q cycle, the heme with the lower potential must correspond to h*b*_l_ of cyt*bc*_1_, which is placed near the Q_p_ site. At this point, however, h*c*_n_ had not been discovered yet; thus, its contribution to the LPC of cyt*b*_6_*f* could not have been estimated.

Studies based on cyt*b*_6_*f* obtained from unicellular algae showed similar results to those based on higher plant enzymes. Flash-induced absorption experiments conducted by Joliot and Joliot corroborated the results of Hurt and Hauska for *E*_m_s of hemes *b* and introduced a new component to the LPC of cyt*b*_6_*f* with *E*_m_ of ∼0 mV.^24^ This new redox component “G”, discovered by Lavergne, is now known to originate from h*c*_n_.^25^

Studies with cyt*b*_6_*f* isolated from *Chlamydomonas reinhardtii* showed different *E*_m_ values for hemes *b* with *E*_m_s of −84 and −158 mV at pH 8.0 for h*b*_n_ and h*b*_p_, respectively.^26^ Further studies performed after the discovery of h*c*_n_ by Alric et al. led to the *E*_m_ values of −150, −50, and +40 mV for h*b*_p_, h*b*_n_, and h*c*_n_, respectively (pH 8.0).^27^ While the values appear in line with those previously reported for higher plant enzymes, Alric et al. stated that there is no evidence allowing for an unambiguous assignment in which heme *b* (high- or low-potential) is located near h*c*_n_ on the negative side of the membrane.

As h*c*_n_ occupies a position of quinone bound at the Q_i_ site of cyt*bc*_1_, the mechanism of the quinone reduction in Q_n_ of cyt*b*_6_*f* might differ from that of the Q_i_ of cyt*bc*_1_. The latter occurs as a sequence of two one-electron transfer steps from h*b*_h_ to the occupant of the Q_i_ site (quinone or semiquinone) and involves a stable semiquinone (SQ) intermediate. An analogical mechanism has long been considered for the Q_n_ site of cyt*b*_6_*f*. However, the close proximity of h*b*_p_ and h*c*_n_ gave rise to an alternative concept that the two-electron reduction of PQ at Q_n_ (PQ_n_) might be a concerted reaction with two electrons coming simultaneously from both these hemes.^2,28^ A difficulty in detecting the SQ in Q_n_ (SQ_n_) of cyt*b*_6_*f* would be in line with this mechanism; however, neither concerted nor sequential reaction has been proven yet. At the same time, recent studies on the mechanism of PQ oxidation at the Q_p_ and Q_o_ sites of cyt*b*_6_*f* and cyt*bc*_1_, respectively, show an unexpectedly stable SQ spin-coupled to a reduced 2Fe2S cluster,^29,30^ the formation mechanism of which remains to be elucidated.

In view of all of these concerns, it is clear that the unambiguous determination of *E*_m_ values and their proper assignments to specific hemes in the LPC of cyt*b*_6_*f* is critical for describing the energy landscape and the mechanism of the catalytic reaction. This requires experimental specificity difficult to achieve for several reasons. First, the resolution of the optical spectra of hemes *b* at room temperature is relatively low. Additionally, the HS h*c*_n_ does not exhibit easily detectable optical components. While in bacterial cyt*bc*_1_, the spectral components originating from individual hemes *b* can be identified by specific mutations designed to change the spectral properties of a particular cofactor; such protein engineering is not feasible in the case of plant cyt*b*_6_*f*. Electron paramagnetic resonance (EPR) spectroscopy is generally more selective in the detection of the heme species, but equilibrium redox titrations and further detection by low-temperature EPR spectroscopy require several milliliters of isolated protein at a concentration of a few tens of micromoles per liter, which have not been achieved so far. Also, a knowledge of the origin of EPR transitions is needed to determine the particular redox-active centers.

In this work, to achieve the experimental specificity required for unambiguous determination of the *E*_m_ values of the hemes of the LPC of cyt*b*_6_*f*, we combined three independent spectroscopic methods of detection of the amount of the reduced species in isolated cyt*b*_6_*f* as a function of the ambient redox potential *E*_h_. Low-temperature optical spectrophotometry and continuous wave (CW) EPR spectroscopy were used to record the spectra of the hemes, while pulse EPR spectroscopy allowed us to monitor the distance-dependent phase relaxation enhancement of the Rieske cluster (2Fe2S) by the oxidized heme components.^31^ The latter method provided information not only on the redox states of the hemes but also on the spatial arrangement of the species of particular redox potential.

Our results indicate that, contrary to the current notion on the *E*_m_s of hemes *b* in cyt*b*_6_*f*, the *E*_m_ value of h*b*_p_ is larger than that for h*b*_n_. This introduces an uphill step for ET in the LPC of cyt*b*_6_*f*, so far never considered in the energy landscape for the catalytic reaction. The mechanistic consequences of this finding are discussed.

## Experimental Section

Cyt*b*_6_*f* was isolated from spinach leaves using a large-scale protocol that was based on protocols of Baniulis^32^ and Romanowska.^33^ Briefly, 10 kg of spinach leaves were homogenized using a whole slow juicer. After filtration and centrifugation (17 000 g, 20 min, 4 °C), the pellet was resuspended in low-ionic-strength buffer and pressed through a French Press, followed by centrifugation (5000 g, 10 min, 4 °C). The supernatant was ultracentrifuged (148 000 g, 30 min, 4 °C), and the resulting pellet containing thylakoid membranes was resuspended in buffer to obtain a 1.5 mg/mL final chlorophyll concentration. Then, the thylakoids were solubilized by adding an equal volume of octyl glucoside solution to a final concentration of 25 mM. After ultracentrifugation (148 000 g, 20 min, 4 °C), the supernatant was collected and applied to a propyl-Sepharose column. The hydrophobic chromatography step was repeated. Cyt*b*_6_*f* eluted from the column was further purified by ultracentrifugation on a continuous sucrose gradient. This procedure yielded 6 mL of pure cyt*b*_6_*f* at a concentration of ∼50 μM. A more detailed description of the isolation procedure is provided in the Supporting Information. Cyt*bc*_1_ was isolated from *Rhodobacter capsulatus* strain as described in ref (34).

Equilibrium redox titration of both cyt*b*_6_*f* and cyt*bc*_1_ was performed in the dark as generally described in ref (35). About 6 mL of 50 μM purified cyt*b*_6_*f* (bicine buffer, pH 8.0) was redox-poised at different *E*_h_s, under anaerobic conditions, by injections of small aliquots of sodium dithionite. At selected *E*_h_, ∼ 300 μL of the sample was withdrawn through a flat optical cuvette, and the solution was subsequently put into argon-flushed EPR quartz Q band and X band tubes, which were then immediately frozen in liquid nitrogen. To minimize water vapor condensations, the tubes were wiped with ethanol before inserting them into a cryostat.

Optical spectroscopy was performed using a home-built spectrophotometer consisting of a Hamamatsu L10290 light source (Hamamatsu L10290), a 0.05-nm-resolution monochromator (Optel M250), a photomultiplier powered by Biologic PMS250, and a homebuilt Arduino-based analog/digital converter. Samples were placed in flat tubes with I.D. of 0.4 mm × 4 mm (CM Scientific Ltd.) and inserted into an ESR900 cryostat, with temperature controlled by an ITC503S instrument (Oxford Instruments). Spectra were recorded with a home-written LabVIEW program at a resolution of 0.1 nm between 500 and 600 nm. Each wavelength point was averaged 8000 times.

CW EPR spectra were measured at 10 K on a Bruker Elexsys E580 spectrometer operating at X band, equipped with a SuperHQ resonator and an ESR900 cryostat. Measurement parameters were as follows: microwave frequency/power, 9.39 GHz/6.45 mW; modulation amplitude, 15 G; and sweep time/width, 671 s/4495 G.

Pulse EPR measurements were performed on a Bruker Elexsys E580 spectrometer operating at Q band (33.58 GHz) at 14 K as described in.^31^ The resonator ER5107/D2/0501 inserted in a CF935 helium cryostat (Oxford Instruments) was used, and the temperature was controlled using an ITC503 temperature controller unit. Electron spin echo decay curves (ESE DCs) were measured at *g* ∼ 1.90, i.e., at the field position of the maximum amplitude of the echo-detected EPR spectrum of the 2Fe2S cluster. This position was selected due to the fact that it encompassed the largest population of 2Fe2S orientations with respect to the external magnetic field and provided the highest signal-to-noise ratio. Each ESE DC was measured using a π/2–*t*–π sequence, with a length of π/2 pulse of 16 ns. The microwave power was adjusted to obtain the maximum amplitude of the echo. The resonator dead time was 440 ns, and prior to analysis, the time axes for each ESE DC were shifted forward by the addition of the dead time. Further, ESE DCs were fitted with a single exponent function, and the fits were extrapolated to zero time to obtain amplitudes of the echo at the onset of the relaxation process. Then, amplitudes at zero time were used to normalize each ESE DC.

## Results and Discussion

For clarity of the presentation of the results, we use terms h*b*_n_ and h*b*_p_ specifically for hemes *b* of cyt*b*_6_*f* according to their position in the structure (i.e., h*b*_n_ is close to Q_n_ and the n-side of the membrane, while h*b*_p_ is close to Q_p_ and the p-side of the membrane). The terms h*b*_h_ and h*b*_l_ are reserved for respective hemes *b* of cyt*bc*_1_. We emphasize upfront that, to explain the ensemble of the spectral and redox properties of hemes presented in this work, we had to abandon the long-standing notion about h*b*_n_ having higher redox potential than h*b*_p_.

### Resolving Optical Components of Low-Spin Hemes in cyt*b*_6_*f* and cyt*bc*_1_

The optical spectra of isolated cyt*b*_6_*f* and cyt*bc*_1_ were measured at different external redox potentials (*E*_h_s) in the 500–600 nm wavelength range. To obtain a better spectral resolution of the components, all spectra were measured at 9 K. At the highest investigated *E*_h_, the heme *f* (h*f*) in cyt*b*_6_*f* and heme *c*_1_ (h*c*_1_) in cyt*bc*_1_ were already fully reduced and thus were treated as constant components present in all of the analyzed spectra. The gradual lowering of *E*_h_s led to the appearance of spectral components originating from the reduced low-spin (LS) h*b*_n_ and h*b*_p_ in cyt*b*_6_*f* and LS h*b*_h_ and h*b*_l_ in cyt*bc*_1_. A significant overlap of these components, especially in cyt*b*_6_*f*, precluded direct determination of the changes in amplitudes of these hemes as a function of *E*_h_; therefore, the global analysis fit (GAF) procedure was applied to find a minimum number of independent components that could reproduce the experimental spectra of both cyts (see details in the Supporting Information (SI)). The optimized parameters describing the shapes of spectral components of LS hemes *b* in cyt*b*_6_*f* and cyt*bc*_1_ are given in Table 1.

**Table 1:** Optical Components for Hemes *b* in cyt*b*_6_*f* and cyt*bc*_1_ at 9 K^a^

heme	analytical function of shape	global parameters of the Gauss functions
cytochrome *b*_6_*f*		
*b*_p_		λ_1bp_ = 557.342 ± 0.020 nm
		σ_1bp_ = 1.49 ± 0.011 nm
		λ_2bp_ = 562.175 ± 0.018 nm
		σ_2bp_ = 1.2809 ± 0.0086 nm
		*r =* 1.4446 ± 0.0078
*b*_n_		λ_1bn_ = 559.952 ± 0.019 nm
		σ_1bn_ = 1.1216 ± 0.021 nm
cytochrome *bc*_1_		
*b*_l_		λ_1bl_ = 553.910 ± 0.038 nm
		σ_1bl_ = 1.363 ± 0.035 nm
		λ_2bl_ = 562.477 ± 0.020 nm
		σ_2bl_ = 1.666 ± 0.023 nm
		*r* = 1.547 ± 0.043
*b*_h_		λ_1bh_ = 556.753 ± 0.011 nm
		σ_1bh_ = 1.768 ± 0.011 nm

a *A*_*x*_ is the local parameter describing the amplitude of the component *x*; λ_*x*_, σ_*x*_, and *r* are global parameters describing the position of maximum absorbance, the half-width of the absorption maxima, and the amplitude coefficient of the longer-wavelength peak of two-Gaussian spectra, respectively.

**Figure 1:**
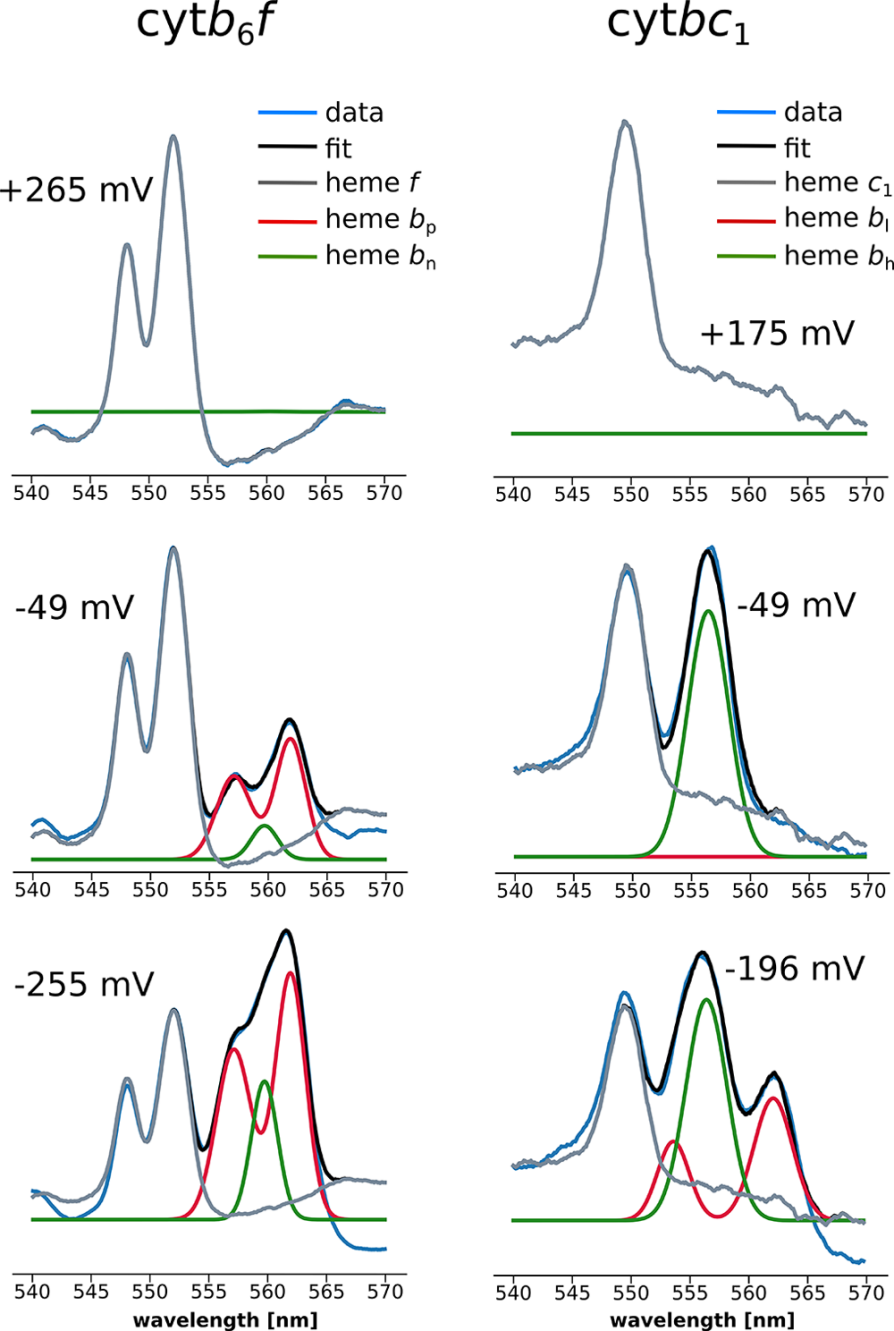
Optical spectra of the LS hemes of cyt*b*_6_*f* (left column) and cyt*bc*_1_ (right column) measured at 9 K for the samples redox-poised at the high (top), intermediate (middle), and low (bottom) external redox potential *E*_h_. The experimental spectra (blue) were fitted with a sum of the respective spectral components (black). Gray lines show the components originating from h*f* and h*c*_1_. Hemes *b*_p_ and *b*_l_ are shown in red, while hemes *b*_n_ and *b*_h_ are shown as green lines. The values indicate the *E*_h_ of the particular sample measured.

The experimental spectra and the emerging components in cyt*b*_6_*f* and cyt*bc*_1_ at the high (+265 and +175 mV, respectively), intermediate (−49 mV), and low *E*_h_s (−255 mV) are shown in Figure 1. It is clear that within the investigated wavelength and *E*_h_ ranges, the optical spectra could be reproduced assuming the presence of LS hemes only. Although cyt*b*_6_*f* contains an additional high-spin (HS) h*c*_n_, we did not identify a contribution that could be ascribed to this heme in this wavelength range. Comparison of the spectral shapes determined by GAF for hemes *b* revealed that h*b*_p_ in cyt*b*_6_*f* is similar to h*b*_l_ in cyt*bc*_1_; these two hemes required use of a two-Gaussian function with similar relative amplitudes of absorption peaks, albeit the positions and the widths of the peaks were different. The h*b*_n_ in cyt*b*_6_*f* showed qualitative similarity to h*b*_h_ in cyt*bc*_1_, and they were both reproduced by a single Gauss function, although the peak positions and widths of the bands were different. The presence of two types of components (one asymmetrical and split and one symmetrical) is in good agreement with previous observations of Hurt and Hauska.^21^

**Figure 2:**
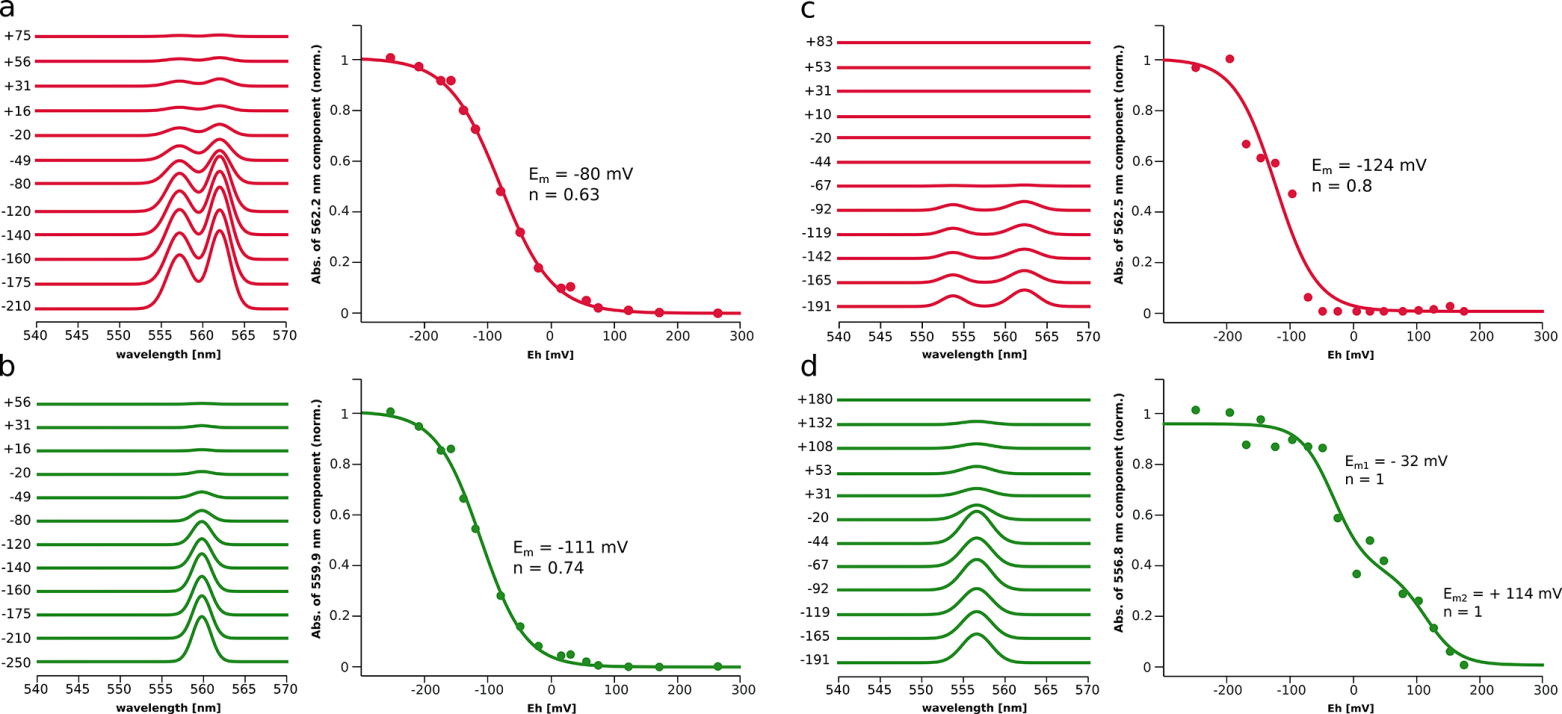
Equilibrium redox titrations of LS hemes of cyt*b*_6_*f* (a, b) and cyt*bc*_1_ (c, d) obtained by decomposition of the optical spectra. (a) Spectral component of hems *b*_p_ in cyt*b*_6_*f* at different *E*_h_s (left), and the Nernst curve fitted to the reduced fraction of the heme (right). (b) Same data as in (a) but for heme *b*_n_. (c) and (d) show the same type of analysis as in (a) and (b) but for hemes *b*_l_ and *b*_h_ of cyt*bc*_1_. Statistical analysis based on confidence intervals is described in the SI (Figure S1).

Having defined the optical components, we examined the change of the amplitudes of hemes *b* as a function of *E*_h_ to obtain their respective *E*_m_s. The results of the fitting of the Nernst curves to the separated optical components are shown in Figure 2. The components ascribed to h*b*_p_ and h*b*_n_ (see Table 1) could be fitted with a single Nernst function yielding −80 mV, *n* = 0.63 and −111 mV, *n* = 0.74, respectively. A similar analysis performed for cyt*bc*_1_ yielded *E*_m_ of h*b*_l_ of −124 mV, *n* = 0.8, while h*b*_h_ exhibited the Nernst curve split into two fractions both with *n* = 1. The first fraction, contributing to 60% of total h*b*_h_, has *E*_m_ = −32 mV, while the remaining 40% fraction has *E*_m_ = +114 mV. Such a split in h*b*_h_ is well defined and described as an inherent feature of this heme.^36−38^

The results obtained from the optical redox titrations of cyt*bc*_1_ comply with the literature data. At the same time, the results obtained for cyt*b*_6_*f* suggested that h*b*_p_, which in the structure occupies a position corresponding to that of h*b*_l_ of cyt*bc*_1_, has higher *E*_m_ than h*b*_n_. This means that the redox potentials of h*b*_p_ and h*b*_n_ in cyt*b*_6_*f* are in a reverse relationship compared to their counterparts h*b*_l_ and h*b*_h_ in cyt*bc*_1_. Therefore, the optical spectrum of LS heme *b* in cyt*b*_6_*f*, which appears as a first component upon lowering *E*_h_, originates from h*b*_p_ and not h*b*_n_. This implies an incorrect assignment of the spectra of these hemes in the literature.

Of note, the *n* parameters obtained for hemes *b* in cyt*b*_6_*f* and h*b*_l_ in cyt*bc*_1_ are significantly lower than 1. This does not mean that the number of electrons involved in the redox reactions is not an integer. It suggests the presence of Coulombic interactions between closely separated hemes where the reduction state of one of the heme influences the redox potential of the other and vice versa.^39^ If *E*_m_s of the hemes are separated by less than approximately 120–140 mV, such interactions may be responsible for effective *n* less than 1 and the apparent stretching of the Nernst curves. Given the distances between hemes in the structures and differences in *E*_m_s, one would expect significant interactions between the following heme *b* pairs: h*b*_n_–h*b*_p_ and h*b*_p_–h*b*_p_ in cyt*b*_6_*f* and h*b*_l_–h*b*_l_ in cyt*bc*_1_.^40^ This indeed was observed in our experiments, and in the case of the h*b*_l_–h*b*_l_ pair, also reported in the literature.^41^

### Redox Titrations of cyt*b*_6_*f* and cyt*bc*_1_ Analyzed by CW EPR Spectroscopy

All of the samples measured by optical spectroscopy were subsequently measured by CW EPR spectroscopy. In the titration analysis, we considered the following EPR transitions. In the case of cyt*bc*_1_, we used well-defined *g* = 3.78 and 3.43, corresponding to LS highly axial low-spin (HALS) oxidized h*b*_l_ and h*b*_h_, respectively. In the case of cyt*b*_6_*f*, we used *g* = 3.65 ascribed to the *g*_z_ transition of the oxidized LS, HALS h*b*_p_.^42^ As h*b*_n_ does not show a separate, clearly identifiable transition, we chose *g* = 12.4 and 4.73 from the signals at *g* > 4.3 ascribed to h*c*_n_ spin-coupled to h*b*_n_^42,43^ (see the SI for a more detailed description of the EPR spectrum of cyt*b*_6_*f*) to monitor the redox-dependent changes in the amplitude of the h*c*_n_–h*b*_n_ pair.

**Figure 3:**
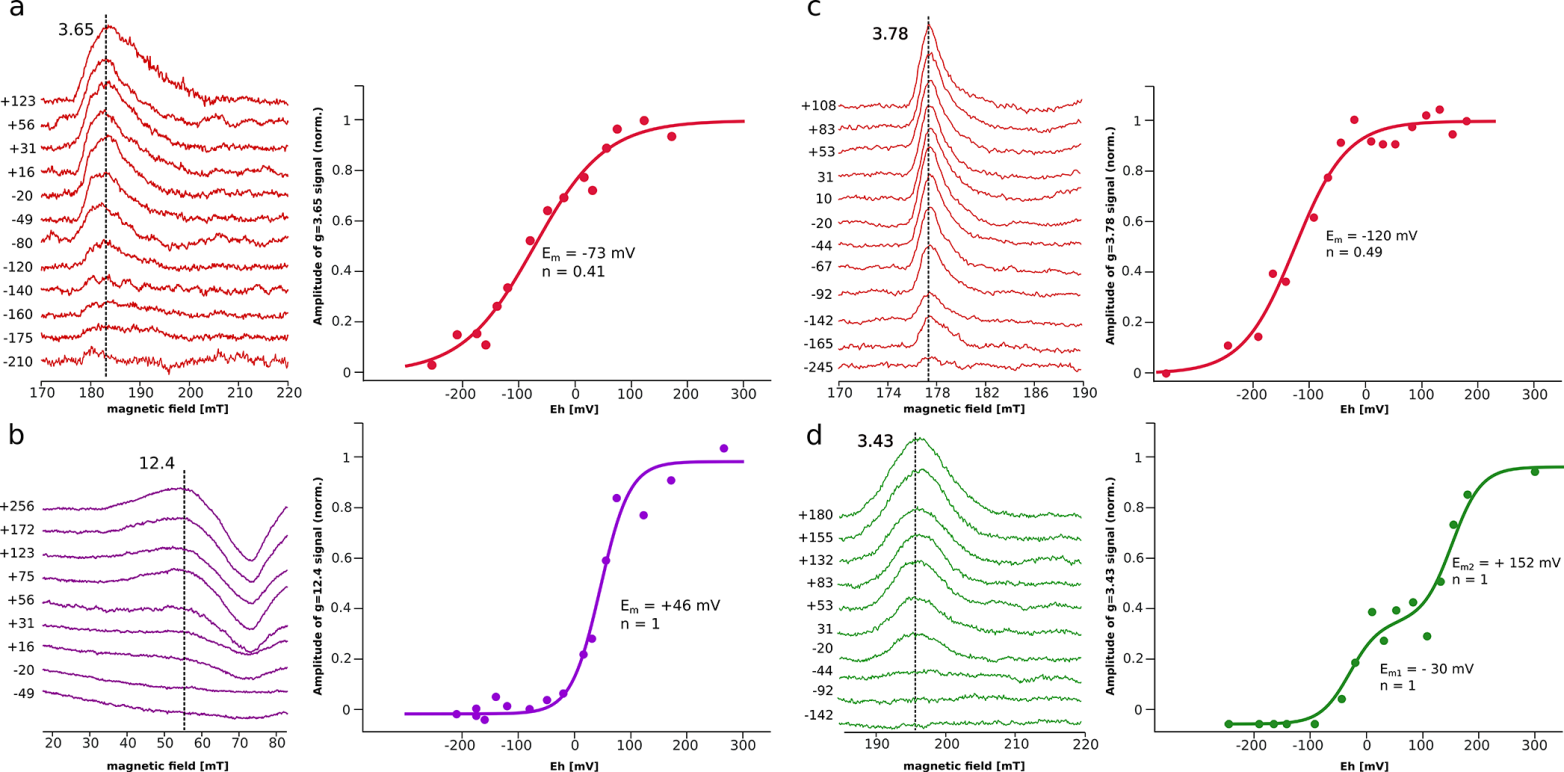
Equilibrium redox titrations of paramagnetic species by CW EPR spectroscopy at 10 K and different *E*_h_s. (a) Spectra of the oxidized h*b*_p_ of cyt*b*_6_*f* (left) and its respective Nernst curve (right). (b) Low-field transition (*g* = 12.4) of oxidized h*c*_n_ spin-coupled to h*b*_n_ (left) and its respective Nernst curve (right). (c, d) show similar analysis for spectra of oxidized h*b*_l_ and h*b*_h_ in cyt*bc*_1_, respectively. Statistical analysis based on confidence intervals is described in the SI (Figure S2).

The amplitude of the signal at *g* = 3.65 gradually decreased upon lowering *E*_h_ (Figure 3a). A fit of the Nernst function to the data yielded *E*_m_ = −73 mV and *n* = 0.41. We note that this transition is relatively weak, and a contribution of background is likely to introduce uncertainties, especially in the low *E*_h_ range, causing an additional decrease in the *n* parameter. Nevertheless, the *E*_m_ value is in good agreement with the *E*_m_ of −80 mV obtained for h*b*_p_ from the optical titration (Figure 2a).

The *g* = 12.4 (and 4.73) signals were taken to construct the Nernst curve of either h*c*_n_ or h*b*_n_, since the reduction of at least one of these hemes in a coupled pair is expected to abolish this signal (Figure 3b). The analysis revealed that the *g* = 12.4 signal decreased upon lowering *E*_h_, and the determined *E*_m_ value for this species was +46 mV, *n* = 1. As there is no optical spectra component of a LS heme species having such a large *E*_m_, we concluded that the disappearance of *g* = 12.4 and 4.73 signals resulted solely from the one-electron reduction of h*c*_n_ and not h*b*_n_. This *E*_m_ value for h*c*_n_ is in good agreement with the literature data.^27^ We note that the species with *E*_m_ of around −111 mV (as determined for h*b*_n_ by optical spectroscopy) was not identified in our CW EPR experiments, confirming the notion that this heme does not show a separate transition. Overall, CW EPR spectroscopy identified two *E*_m_ values of −73 and +46 mV, corresponding to h*b*_p_ and h*c*_n_, respectively.

Fitting the Nernst curve to the data of HALS *g* = 3.78 and 3.43 signals in cyt*bc*_1_, originating from h*b*_l_ (Figure 3c) and h*b*_h_ (Figure 3d), respectively, yielded results consistent with data from optical spectroscopy. The amplitude of the EPR signal of h*b*_l_ decreased upon lowering *E*_h_, and the Nernst curve yielded *E*_m_ = −120 mV, *n* = 0.49. In the case of h*b*_h_, the EPR signal also decreased upon lowering *E*_h_ and, similar to the optical data, there were two redox fractions detected with *E*_m_ +152 and −30 mV, both with *n* = 1.

Redox properties of hemes *b* determined by optical and CW EPR spectroscopies for both cyt*b*_6_*f* and cyt*bc*_1_ are summarized in Table 2.

**Table 2:** Redox Properties of Hemes *b* in cyt*b*_6_*f* and cyt*bc*_1_ Determined by Optical and EPR Spectroscopies

heme	*E*_m_ [mV] (optical spectroscopy)	*E*_m_ [mV] (CW EPR)
cytochrome *b*_6_*f*		
*c*_n_	n.d.	+46 ± 5, *n* = 1.02 ± 0.17
*b*_p_	–80 ± 2, *n* = 0.63 ± 0.02	–73 ± 6, *n* = 0.41 ± 0.04
*b*_n_	–111 ± 2, *n* = 0.74 ± 0.04	n.d.
cytochrome *bc*_1_		
*b*_l_	–124 ± 6, *n* = 0.80 ± 0.13	–120 ± 6, *n* = 0.49 ± 0.06
*b*_h_	–32 ± 19, *n* = 1.0 ± 0.5	–30 ± 20, *n* = 1.0 ± 0.7
	+114 ± 30, *n* = 1.0 ± 0.7	+152 ± 13, *n* = 1.0 ± 0.5

### *E*_h_-Dependent Enhancement of the Phase Relaxation of 2Fe2S in cyt*b*_6_*f* and cyt*bc*_1_

In addition to the analysis of redox titration data for cyt*b*_6_*f* and cyt*bc*_1_ obtained by optical and CW EPR spectroscopies, we performed measurements of the ESE DCs of the 2Fe2S cluster at 14 K for samples poised at different *E*_h_s.

**Figure 4:**
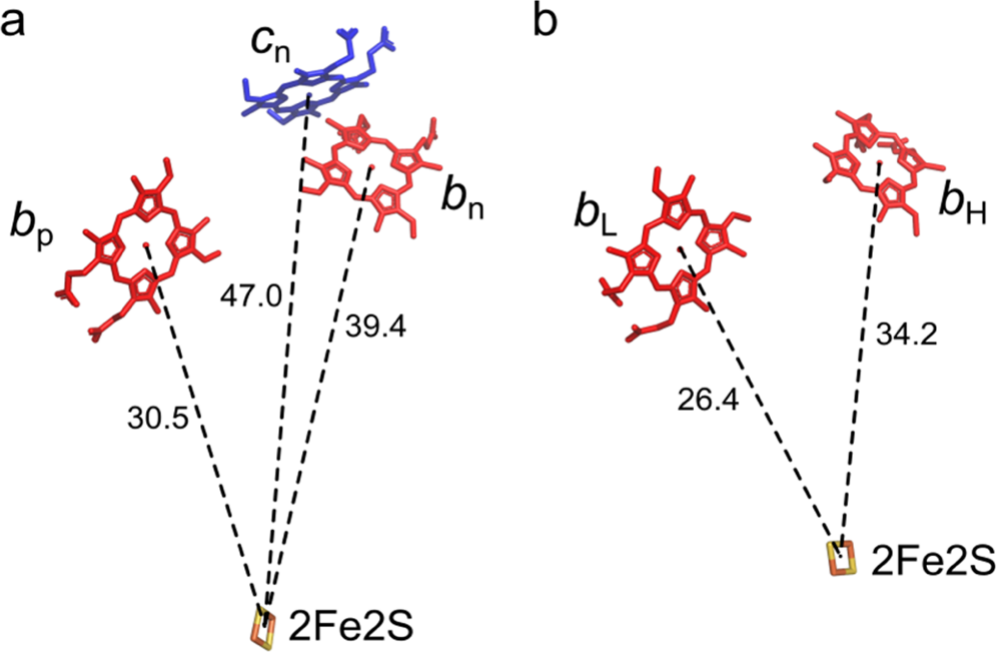
Comparison of spatial arrangement of 2Fe2S and hemes in monomers of cyt*b*_6_*f* (a) and cyt*bc*_1_. The LS hemes are shown as red sticks, while the HS h*c*_n_ in *a* is shown as blue sticks. Dotted black lines show the distances between the 2Fe2S cluster (orange-yellow sticks) and heme iron ions. The distances were measured on the basis of the PDB structures of cyt*b*_6_*f* (ID:6RQF) and cyt*bc*_1_ (ID:1ZRT).

Within the investigated *E*_h_ range, 2Fe2S and h*f* of cyt*b*_6_*f* remained fully reduced and had *S* = 1/2 and *S* = 0 spin states, respectively. The other hemes changed their spin states upon reduction from *S* = 1/2 to *S* = 0 (hemes *b* in cyt*b*_6_*f* and cyt*bc*_1_) or from *S* = 5/2 to *S* = 2 (h*c*_n_ in cyt*b*_6_*f*). Since ESE decay rates depend on the strength of dipolar interactions (defined by average dipolar relaxation time constants *T*_dip_) with fast-relaxing paramagnetic centers in a distance-dependent manner,^44−46^ we expected to observe a gradual slowing down of the phase relaxation of 2Fe2S upon reduction of hemes *b* and h*c*_n_ with decreasing *E*_h_.^31^Figure 4 shows distances between 2Fe2S and hemes *b* and h*c*_n_ in a monomer of cyt*b*_6_*f* and cyt*bc*_1_. Clearly, h*b*_p_ or h*b*_l_ is positioned closer to 2Fe2S than h*b*_n_ or h*b*_h_. Therefore, in the most simplified case, it can be anticipated that h*b*_p_ or h*b*_l_ should exert a stronger effect on the relaxation of 2Fe2S as defined by shorter *T*_dip_ compared to h*b*_n_ or h*b*_h_.

Performing GAF for ESE DCs (see details of the model, basic assumptions, and analytical approach in the SI) to estimate *T*_dip_ values provided a crude approximation of the distance-dependent enhancement of the phase relaxation of 2Fe2S induced by dipolar interactions with hemes *b* in cyt*b*_6_*f* and cyt*bc*_1_ and h*c*_n_ in cyt*b*_6_*f*. In the case of cyt*b*_6_*f*, we used nine global adjustable parameters: three representing *T*_dip_ values and six representing the parameters of the Nernst curves (*E*_m_ and *n*). Fixing *n* parameters to those obtained from optical and CW EPR spectroscopies led to instability of the fit; thus, estimated *T*_dip_ and particularly *E*_m_ values were not sensible due to the stretching of the Nernst curves used for fitting. This effect caused the mixing of the contributions from different species to the relaxation enhancement of 2Fe2S. Therefore, we simplified GAF by fixing *n* values to 1 for all of the species. The results of the GAF of the ESE curves are shown in Table 3.

**Table 3:** Dipolar Decay Time Constants and *E*_m_ Determined from Pulse EPR Spectroscopy of 2Fe2S in cyt*b*_6_*f*

heme	*T*_dip_ [μs] (14 K)	*E*_m_ [mV], *n* = 1
*c*_n_	1.6	19
*b*_p_	2.2	–95
*b*_n_	3.4	–145

Application of GAF to ESE DCs included three different species that may contribute to the enhancement of phase relaxation of 2Fe2S with *E*_m_ values optimized at +19, −95, and −144 mV, with the strongest, intermediate, and the weakest impact on the relaxation, respectively. Although the *E*_m_ values obtained from ESE DC seem approximately 20–30 mV lower than those from optical and CW EPR spectroscopies, they generally stay in agreement with the spectral analysis. The relatively small differences in *E*_m_s may result from several experimental limitations of ESE measurement and the simplified model used for GAF. Therefore, the results, especially in regard to *T*_dip_, should be treated only as quasi-quantitative approximations of dipolar enhancement of the 2Fe2S relaxation. Comparison of *E*_m_s obtained from pulse EPR spectroscopy to data from optical and CW EPR spectroscopies suggests that the strongest effect on relaxation results from interactions of the cluster with h*c*_n_, an intermediate for h*b*_p_, and the weakest for h*b*_n_. Despite the fact that HS h*c*_n_ is the most remote from 2Fe2S, its influence can be the strongest, as its magnetic moment and spin-lattice relaxation rate are the highest among paramagnetic hemes in cyt*b*_6_*f*.^47,48^ Additionally, after the reduction, this heme is not converted into a diamagnetic species. Conversely, both h*b*_n_ and h*b*_p_ paramagnetic centers are more similar to each other than to h*c*_n_; they are LS hemes and undergo conversion to diamagnetic species upon reduction, completely abolishing their effects on the 2Fe2S relaxation. Hence, we assume that the effect of h*b*_p_ and h*b*_n_ on 2Fe2S is more directly associated with the distance to the cluster. The results shown in Table 3 suggest that in fact h*b*_p_, for which *E*_m_ determined from pulse EPR is ∼ −90 mV, is closer to 2Fe2S than h*b*_n_ for which *E*_m_ is lower.

To verify this conclusion we performed analogous experiments on cyt*bc*_1_ for which *E*_m_s and spectral properties are well defined. The results of GAF for ESE DCs measured at 14 K in cyt*bc*_1_ provided an estimation of *T*_dip_, when *E*_m_ values obtained from optical spectroscopy were fixed at −120 mV for h*b*_l_ and for h*b*_h_ at −30/+120 mV (with a contribution of 60/40%, respectively). For h*b*_l_, *T*_dip_ is ∼0.82 μs, while for the more distant h*b*_h_, it is ∼1.38 μs. Such semiquantitative results for cyt*bc*_1_ are expected, considering that these hemes have similar paramagnetic properties and h*b*_l_ is closer to 2Fe2S than h*b*_h_. It generally shows that the species closer to 2Fe2S exerts a stronger effect on its relaxation. Thus, a comparison of the results obtained for cyt*b*_6_*f* to those for cyt*bc*_1_ seems to justify the conclusion that in fact the LS heme species in cyt*b*_6_*f* with the lowest *E*_m_ is more distant from 2Fe2S. Based on the spatial arrangement of hemes in cyt*b*_6_*f* shown in Figure 4, this must be h*b*_n_.

### Mechanistic Consequences of an Uphill Step in the LPC of cytb_6_f

**Figure 5:**
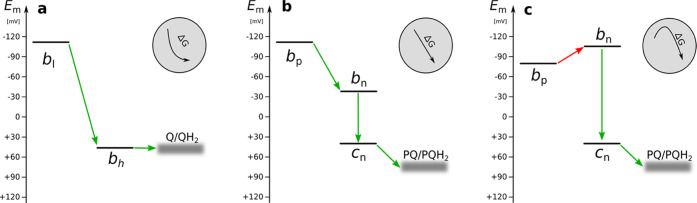
Energy landscapes of LPCs in cyt*bc*_1_ (a) and cyt*b*_6_*f* (b, c). (a) and (c) show energy landscapes resulting from this study, while (b) shows energy landscapes previously assumed for cyt*b*_6_*f*. Energies are expressed in midpoint redox potentials of hemes. Green arrows indicate downhill steps, while the red arrow indicates the newly established uphill step in the energy profile of cyt*b*_6_*f*. Gray inlets show the general profiles of the energy landscapes.

The proximity of h*c*_n_ to h*b*_n_ and the difficulty in detecting SQ at Q_n_ suggested the intriguing new possibility that the two-electron PQ reduction at the Q_n_ site might follow the concerted mechanism rather than a sequential mechanism long considered in analogy to the well-defined reaction of ubiquinone reduction at the Q_i_ site of cyt*bc*_1_ (Figure 5a). In the concerted reaction, the two electrons are assumed to reduce PQ simultaneously without the formation of a SQ intermediate. As these electrons would have to come from h*c*_n_ and h*b*_n_, the concerted reaction is primed by the state in which both these hemes are reduced (Figure 5b). However, our titration results indicate that the occupancy of this state is much lower than that originally assumed: if two electrons are available in the LPC, they will preferably reside on h*c*_n_ and h*b*_p_ rather than on h*c*_n_ and h*b*_n_, due to the lowest *E*_m_ of the latter (Figure 5c). This in itself does not dismiss the concerted electron transfer to PQ_n_, as the reaction sequence would largely depend on the stability of SQ_n_. If SQ_n_ is stable (i.e., the *E*_m_ values of PQ_n_/SQ_n_ and SQ_n_/PQH_2_ couples are similar,^49^ assuming that the *E*_m_ of PQ_n_/SQ_n_ is lower), the energy landscape proposed in this work favors the sequential mechanism of PQ_n_ reduction due to spatial separation of two electrons on the LPC. However, if SQ_n_ is unstable (i.e., large separation between *E*_m_s of PQ_n_/SQ_n_ and SQ_n_ /PQH_2_ pairs, still, assuming that *E*_m_ of the first one is lower), the low *E*_m_ of h*b*_n_ might in fact favor initiation of the PQ_n_ reduction, which would proceed rapidly in a concerted reaction. At present, the stability of SQ_n_ is unknown and therefore discriminating between these two scenarios is not possible.

Regardless of the quinone reduction mechanism at the Q_n_ site, the uphill step at the level of h*b*_n_ is likely to affect electron distribution in the LPC^41^ and slow down electron transfer from the Q_p_ site through the LPC to the Q_n_ site. Based on the Moser–Dutton ruler,^50^ the estimated rate of the ET from h*b*_n_ to PQ_n_ in the classical scheme (all downhill reactions) would be of the order of 10^6^ s^–1^. The presence of the uphill step reduces this rate to 10^5^ s^–1^. This shift in the estimated ET rate might be of physiological significance. Photosynthesis in plants involves two electron transfer paths that need to be appropriately balanced: a linear electron flow (LEF), which links two photosystems through cyt*b*_6_*f*, and a cyclic electron flow (CEF), which reduces PQ using electrons introduced from the N-side of the membrane. The electron transfer from Q_p_ to Q_n_ sites, as part of the catalytic cycle of cyt*b*_6_*f*, is an inherent step of LEF. If h*c*_n_ provides an entry for the electrons in CEF to reduce PQ at Q_n_ (as some models assume^16,17^), it is reasonable to assume that the uphill step at the level of h*b*_n_ might provide an important element of regulation of the rate of CEF vs LEF in photosynthesis. Assuming that CEF involves ET from a potential n-side electron donor (possibly ferredoxin) to PQ_n_, the rate of this reaction (considering the closest possible distance from the ferredoxin iron–sulfur cluster to PQ_n_ and their redox potentials) would be of the order of 10^5^–10^6^ s^–1^. As this estimated rate is comparable to the rate of the ET through the LPC, an uphill step could be seen as an adaptation of the LPC to facilitate both LEF and CEF.

Another consequence of an uphill step in LPC is that electrons might tend to reside at the h*b*_p_/Q_p_ site for a prolonged time, which in turn might increase the probability of the formation of SQ radicals at this site.^51,52^ Indeed, a SQ spin-coupled to 2Fe2S was observed in native, noninhibited cyt*b*_6_*f* during catalysis.^30^ To observe such a state in cyt*bc*_1_, which has no uphill step in LPC, it is necessary to add a Q_i_ site inhibitor that blocks electron transfer through LPC.

## Conclusions

Application of three independent and comprehensive low-temperature spectroscopies (continuous wave and pulse EPR and optical spectroscopies) in equilibrium redox titrations of cyt*b*_6_*f* resolved a long-standing uncertainty concerning the *E*_m_ values of hemes *b* that support the cross-membrane electron transfer in photosynthesis. Optical spectroscopy identified unambiguously components originating from reduced hemes *b*, revealing that heme *b*_p_ is described by a sum of two while *b*_n_ is by one Gauss function. The component ascribed to heme *b*_n_ is of particular importance given that there is no other reliable indicator of its oxidation state. The components corresponding to heme *b*_p_ and *b*_n_ were titrated at pH 8 with the *E*_m_ values of −80 and −111 mV, respectively.

The *g* = 12.4 transition resulting from spin–spin interactions between oxidized h*c*_n_ and h*b*_n_ disappears upon decreasing *E*_h_ and reports the reduction of h*c*_n_ with *E*_m_ = +44 mV and not the reduction of h*b*_n_. The signal at *g* = 3.65, originating from HALS heme *b*_p_, was found to titrate with *E*_m_ ∼ −73 mV (pH 8), which stayed in line with the *E*_m_ of −80 mV, determined from optical spectroscopy. In accordance with the previous analysis, no EPR transition could be ascribed to h*b*_n_.

Pulse EPR measurements of distance-dependent relaxation enhancement of the phase relaxation of the 2Fe2S cluster revealed that the component originating from heme *b* having higher *E*_m_ is positioned closer to the cluster than that having lower *E*_m_. This result, in view of the known distances of cofactors in the structures of cyt*b*_6_*f*, confirmed that the redox midpoint potential of heme *b*_p_ is higher than the *E*_m_ of heme *b*_n_.

Our conclusion dismisses the long-standing assumption that h*b*_p_ has *E*_m_ lower than that of h*b*_n_, in analogy to the well-established *E*_m_ values of the respective hemes *b* (*b*_l_ and *b*_h_) of homologous cyt*bc*_1_. It is thus clear that the terms h*b*_l_ and h*b*_h_ (where “l” and “h” stand for low and high potential, respectively) hold true only for hemes *b* of cyt*bc*_1_ but not cyt*b*_6_*f*.

The defined order of *E*_m_s implies the existence of an uphill step for electron transfer from heme *b*_n_ to heme *b*_p_. It also indicates that in the spin-coupled pair of hemes *b*_n_ and *c*_n_, the former heme has an approximately 150 mV lower *E*_m_ than the latter. This is expected to impact the probabilities of the occurrence of specific reactions in both the Q_n_ and Q_p_ sites. In the Q_n_ site, it may favor a sequential or concerted mechanism of PQ reduction, depending on the actual stability of SQ_n_. It also slows down the electron transfer from the Q_p_ to Q_n_ site and increases the probability of the formation of SQ_p_. All of these effects help in balancing the rates of the linear and cyclic electron transfer through cyt*b*_6_*f*, thus contributing to the regulation of photosynthetic electron flow.

## Supporting Information Available

Detailed description of the isolation protocol; statistical analysis of data obtained by equilibrium redox titration; EPR spectra of cyt*b*_6_*f*; and the details of the global analysis fit procedure for pulse EPR data (PDF)
