# Supplementary material for: Unexpected Heme Redox Potential Values Implicate an Uphill Step in Cytochrome b6f
Source: J Phys Chem B. 2022 Nov 18;126(47):9771–80. doi: 10.1021/acs.jpcb.2c05729 (PMC9720722; doi:10.1021/acs.jpcb.2c05729)
Supplement: Supplementary file 1 — jp2c05729_si_001.pdf [file jp2c05729_si_001.pdf]

Unexpected Heme Redox Potential Values Implicate an Uphill Step in Cytochrome b_6f

Mateusz Szwalec, Łukasz Bujnowicz, Marcin Sarewicz, Artur Osyczka*

Department of Molecular Biophysics, Faculty of Biochemistry, Biophysics and Biotechnology
Jagiellonian University, Gronostajowa 7, 30-387 Kraków

Abstract: Cytochromes *bc*, key enzymes of respiration and photosynthesis, contain highly-conserved two-heme motif supporting cross-membrane electron transport (ET) that connects the two catalytic quinone-binding sites (Q_n and Q_p). Typically, this ET occurs from the low-to high-potential heme *b*, but in photosynthetic cytochrome b_6f the redox midpoint potentials (E_ms) of these hemes remained uncertain. Our systematic redox titration analysis based on three independent and comprehensive low-temperature spectroscopies (continuous wave and pulse EPR and optical) allowed for an unambiguous assignment of spectral components of hemes in cytochrome b_6f and revealed that E_m of heme b_n is unexpectedly low. Consequently, the cross-membrane ET occurs from the high- to low-potential heme introducing an uphill step in the energy landscape for catalytic reaction. This slows down the ET through low potential chain which can influence the mechanisms of reactions taking place at both Q_p and Q_n sites and modulate efficiency of cyclic and linear ET in photosynthesis.

Table of Contents

Experimental Procedures.. S2
Materials S2
Detailed protocol of cytb_6f isolation S2
Concentration and activity measurements S2
Results and Discussion S3
Statistical analysis S3
Analysis of optical spectra S4
Analysis of EPR spectra S4
Global analysis fit of ESE decay curves S5
Discussion on relaxation enhancement S7
References S7
Author Contributions S8

Experimental Procedures

Materials

Buffers, salts and decylplastoquinone were purchased from Sigma. Detergents UDM and OG were purchased from Glycon (Germany). DDM was purchased from Anatrace. Propyl-Sepharose resin was prepared using activated Sepharose-CNBr powder purchased from Cytiva (Sweden). The activated Sepharose-CNBr was modified through reaction with propylamine (Sigma) according to the manual delivered by the supplier.

Detailed protocol of cytb_6f isolation

Dimeric cytb_6f complex was isolated from market spinach (*S. oleracea*) leaves using a large-scale protocol which was adapted from Baniulis et al.[1] and Romanowska[2]. Spinach leaves were homogenized in buffer 1 (50 mM Tris pH 8, 50 mM NaCl, 200 mM sucrose, 0.2 ml/L antifoam A) using a whole slow juicer. The resulting solution was filtered through a sieve and a must filter. After filtering, the solution was centrifuged at 17 000 g for 20 min, 4°C. The pellet containing chloroplasts was suspended in buffer 2 (10 mM Tris pH 7.5, 10 mM NaCl). Protease inhibitors (benzamidine, ε-amino caproic acid, and PMSF) were added to the chloroplast suspension before it was passed through a French Press (16 000 PSI). The resulting solution was centrifuged at 1000 g for 5 min, 4°C and the supernatant was ultracentrifuged at 148 000 g for 30 min, 4°C. The pellet was resuspended in buffer 3 (30 mM Tris pH 7.5, 50 mM NaCl, 1mM EDTA). The chlorophyll content in the suspension was determined using the protocol described by Porra, R.J. et al.[3]. The volume of the suspension was adjusted to a concentration of 1.5 mg/ml of chlorophyll by dilution with buffer 3. An equal volume of buffer 4 (buffer 3 supplemented with 52 mM octyl glucoside (OG), 0.2 % sodium cholate, and 400 mM ammonium sulfate (AS)) was slowly added to achieve selective solubilization of thylakoid membranes. The solution was subsequently stirred for 15 min and ultracentrifuged at 148 000 g for 20 min, 4°C. The supernatant was brought to 37% of AS saturation and centrifuged at 24 000 g for 10 min, 4°C. Then the supernatant was filtered through a 220 nm membrane and loaded onto a propyl-sepharose column pre-equilibrated with buffer 5 (buffer 3 with 1 mM undecyl α-D-maltoside (UDM) and 37% of AS saturation). The column was washed thoroughly with buffer 5 until the eluent was colorless. A greenish-brown band containing cytb_6f was eluted with buffer 6 (buffer 3 with 1 mM UDM and 20% of AS saturation). The eluent was again brought to 37% of AS saturation and centrifuged at 24 000 g for 10 min, 4°C. After filtering through a 220 nm membrane, the supernatant was loaded onto a second propyl-sepharose column pre-equilibrated with buffer 5. The column was washed stepwise with buffer 5 containing decreasing amounts of AS (saturation decreasing from 35% to 30%), before a brownish band was eluted with buffer 6. The eluate was concentrated using microconcentrators (Merck Millipore) and the buffer was exchanged to buffer 7 (buffer 3 with 1 mM UDM). The sample was loaded onto a 10-25% continuous sucrose gradient in buffer 7 and ultracentrifuged at 141 000 g for 16 hours, 4°C. A brown band in the middle of the gradient containing pure cytb_6f was carefully collected and the buffer was exchanged to buffer 7. Further biochemical analysis (including enzymatic activity, optical and EPR spectroscopy, electrophoretic analysis) indicated that this fraction contained a full complement of the cytb_6f subunits and was enzymatically active.

Concentration and activity measurements

Concentration of cytb_6f was determined spectrophotometrically by measurement of ascorbate-reduced minus ferricyanide-oxidized absorption spectra at 554 nm, relative to isosbestic point 543 nm, using differential extinction coefficient of heme f $\varepsilon_{554\text{-}543} \approx 25\ mM^{-1} cm^{-1}$ [4]. The enzymatic activity of cytb_6f was determined by measuring the cytb_6f-mediated reduction of plastocyanin. Preparation of substrates for activity measurement involved several procedures. Plastocyanin was oxidized with potassium ferricyanide which was subsequently removed by concentration-dilution cycle on microconcentrators. Decylplastoquinone was dissolved in ethanol and

reduced to decylplastoquinol (dPQH$_2$) with hydrogen gas in the presence of platinum on carbon as the catalyst. After reduction, dPHQ$_2$ was diluted 3 times with dimethyl sulfoxide.
The enzymatic reaction was carried out in buffer 3 (from isolation protocol) containing 20 µM of oxidized PC and 100 µM dPQH$_2$. The total volume of the reaction mixture was 1 mL. The enzymatic reaction was initiated by injection of cytb_6f to final concentration of 20 nM (concentration calculated per cyt*f*) and the reaction progress was monitored using a Biologic Diode Array spectrophotometer. The reduction rate of plastocyanin was determined from the initial slope after the addition of cytb_6f to the cuvette. The turnover rate of the sample was estimated to be approximately 120/s. Concentration and enzymatic activity of cytbc_1 was assessed as described in [5].

Results and Discussion

Statistical analysis

Figures S1 and S2 show the statistical analysis of equilibrium redox titration data obtained for cytb_6f and cytbc_1. As it is not possible to precisely assess the uncertainty of each of the external redox potential (E_h) value, it was assumed that the uncertainty is the same for all data points. The maximum value of uncertainty was assumed to be ± 15 mV, which was converted to standard uncertainty (u(E_h) = 8.66 mV) and used to create horizontal error bars.

Figure S1 shows the analysis of the results obtained by decomposition of optical spectra. Due to the fact that standard errors of the amplitudes of reduced hemes were negligible, the points are indicated only as horizontal error bars.

Figure S2 shows the analysis of the results obtained by analysis CW EPR spectra. Vertical error bars represent the uncertainties in determination of the amplitudes of the oxidized hemes. The uncertainty was calculated as standard uncertainty arising from both: the normalization of spectra on the amplitude of 2Fe2S and the noise present in the spectra.

The statistical significance of the model with respect to data was assessed by constructing confidence intervals at confidence level of 95%. Confidence intervals are depicted as blue confidence bands. Values of confidence intervals at each data point were calculated using the expression

$$Y_i = \hat{Y}_i \pm t_{(1-\alpha/2,n-p)} RMSE\sqrt{[1/n + (X_i - X_m)^2/S_{xx}]} \quad \text{eq. S1}$$

where: $\hat{Y}_i$ is the value of fitted function at given *Eh* (X_i), *t* is the 100*(1-α/2) percentage point of Student's *t* distribution on *n-p* degrees of freedom (*n* = number of data points, *p* = number of parameters), RMSE is the Root Mean Squared Error, X_m is the mean of the E_h values and $S_{xx} = \Sigma(X_i - X_m)^2$.

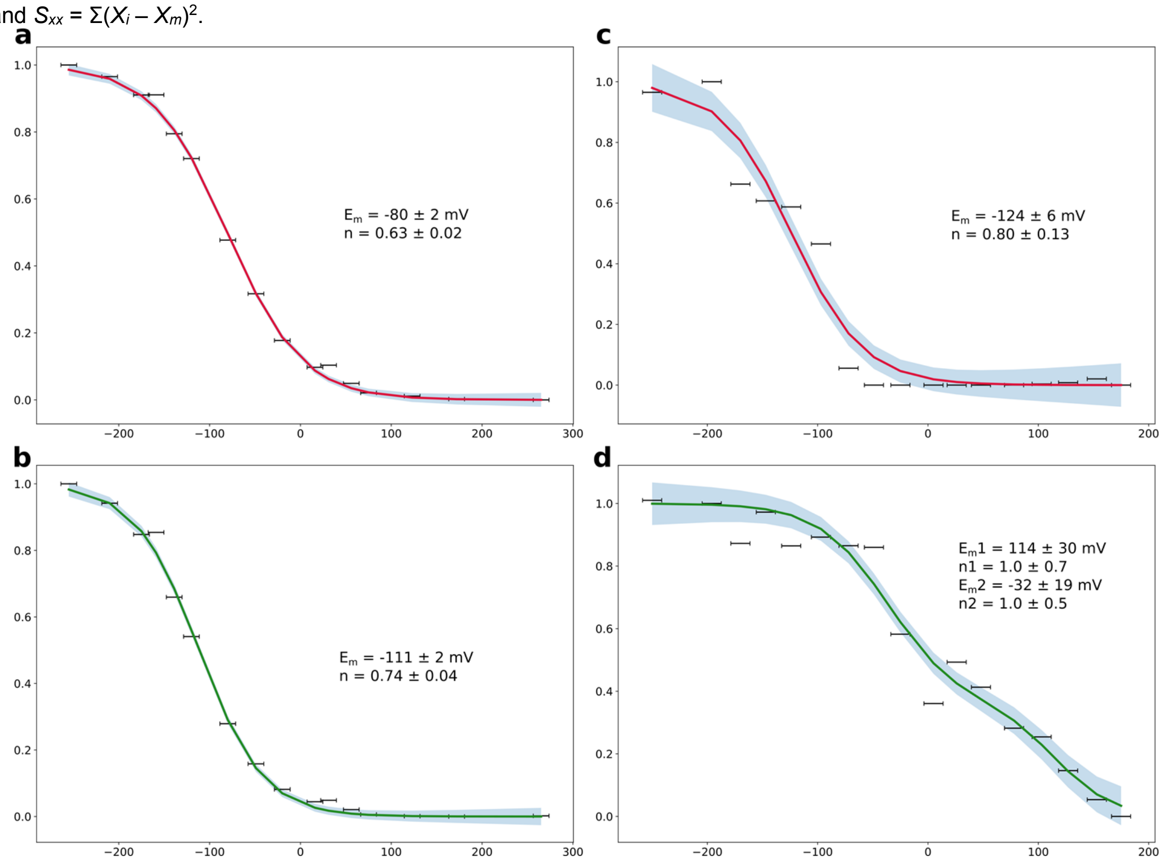

Figure S1. Statistical analysis of the results, shown in Figure 2 in Main Text, obtained by decomposition of the optical spectra. a) and b) show the Nernst curves fitted to the reduced fractions of hemes b_p and b_n of cytb_6f, respectively. c) and d) show the same type of

analysis for hemes b_l and b_h of cyt*bc*$_1$, respectively. Data points are represented only by horizontal error bars for clarity. Data was fitted with the least squares method (Levenberg-Marquardt) using the Nernst function. Fitted values are given with the standard error calculated from the diagonal elements of covariance matrix. Confidence intervals at 95% confidence level are shown as blue bands.

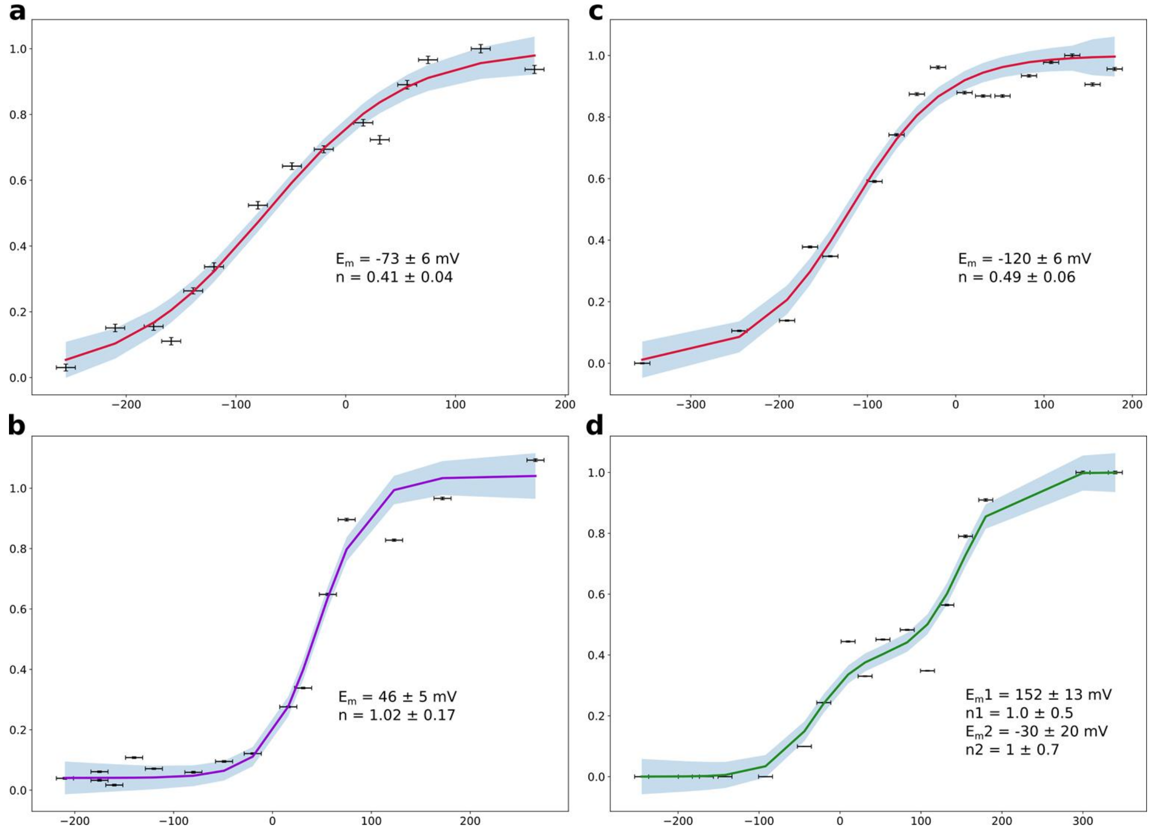

Figure S2. Statistical analysis of the results, shown in Figure3 in Main Text, obtained by analysis of CW EPR spectra. a) and b) show the Nernst curves fitted to the oxidized fractions of hemes b_p and b_n of cyt*b*$_6$*f*, respectively. c) and d) show the same type of analysis for hemes b_l and b_h of cyt*bc*$_1$, respectively. Data points are represented only by error bars for clarity of presentation. Data was fitted with the least squares method using the Nernst function. Fitted values are given with the standard error calculated from the diagonal elements of covariance matrix. Confidence intervals at 95% confidence level are shown as blue bands.

Analysis of optical spectra

Low-temperature optical spectra of cyt*b*$_6$*f* and cyt*bc*$_1$ were analyzed using GAF procedure. The analysis was performed with a self-written Python program utilizing SciPy, NumPy and Pandas packages[6-8]. Each spectrum was fitted with a combination of three components: baseline, two-gaussian function and one-gaussian function. Baseline consisted of a spectrum recorded at high E_h where only h*f* and hc_1 are reduced and a linear function to correct for slope deviation. Two-gaussian component was used to fit hb_p and hb_l while one-gaussian component fit hb_n and hb_h. Other variations of components including any combination of two or more one- or two-gaussian functions were also examined but the best fit was obtained by fitting one two-gaussian component (hb_p and hb_l) and one one-gaussian component (hb_n and hb_h).

Implementation of GAF procedure was achieved by dividing the parameters into two sets: global, that should be held the same for all spectra and local, which could vary from spectrum to spectrum. The set of local parameters consisted of the amplitudes of the components and linear function arguments. The peaks widths, positions and relative amplitude (hb_p and hb_l) were fitted as global parameters, since those values determined the shape of the component and should be the same for all spectra. Model consisting of 3 components with both global and local parameters was fitted to the data matrix using Levenberg-Marquardt algorithm. Reasonable boundary conditions were applied in the fitting procedure. The model fit the data very well as depicted in Figure 1 (Main text). Components for hemes *b* of both cyt*b*$_6$*f* and cyt*bc*$_1$ with optimized values are shown in Table 1 (Main text).

Analysis of EPR spectra

While EPR spectra of cyt*bc*$_1$ has been extensively studied and the EPR transitions of LS hemes *b* have been recognized, the presence of HS hc_n in cyt*b*$_6$*f* creates additional EPR transitions originating from spin-spin exchange interactions with hb_n. Typical CW EPR spectra of cyt*b*$_6$*f* measured at 3 different E_h are shown in Figure S3. A rhombic EPR spectrum at $1.76 < g < 2.02$ originates from the reduced 2Fe2S cluster. The signal of h*f* (g = 3.51) was not detected, as this heme was fully reduced. The transition at g = 3.67 was previously ascribed to g_z transition of the oxidized, highly axial low-spin (HALS) hb_p. The signals at g > 4.3 were ascribed to hc_n which is spin-coupled to hb_n. An additional component at g ~ 6 of unknown species, suggests a presence of a HS heme of axial symmetry. It is likely that it originates from either partially denatured h*f* or a heme *b,* or from a small fraction of hc_n which is not

coupled to hb_n. Similar axial HS signal of heme was detected in cytochrome *c* oxidase, despite the presence of spin-spin exchange interaction with the copper ion.

Well separated EPR signals ascribed to the pair of hc_n and hb_n were found at g = 12.4 and 4.73, thus they were used as a measure of the redox-dependent changes in the amplitude of oxidized hc_n to construct the Nernst curves for this particular pair of hemes.

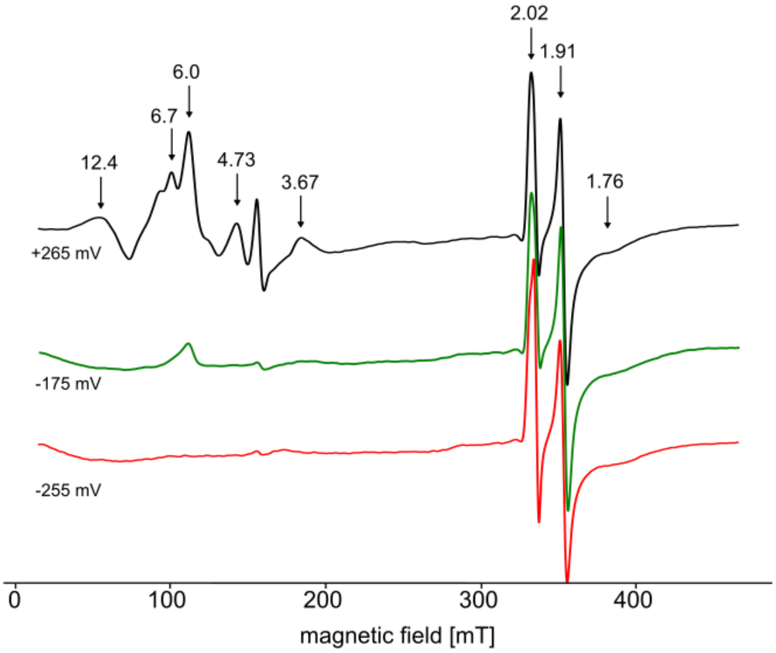

Figure S3. CW EPR spectra of cytb_6f poised at the high (black), intermediate (green) and low E_h. The arrows indicate g values of EPR transitions of the oxidized hemes b_p, (3.67), c_n spin-coupled to heme b_n (4.73, 6.7, 12.4), an axial H-S heme species (6.0) and the reduced 2Fe2S cluster (1.76, 1.91 and 2.02).

Global analysis fit of ESE decay curves

The model used for the global analysis fit of ESE decay curves included following assumptions:

a) only dipolar interactions within a single monomer were considered;
b) a number of fast relaxing species having different E_m in cytb_6f was 3 - hemes b_n, b_p and c_n;
c) dipolar ESE decay curve resulting from the interactions between 2Fe2S and a heme iron was approximated with mono-exponential function described by average T_{dip};
d) effect of changes in the redox state of heme c_n and b_n on ESE decay of 2Fe2S were treated separately, despite existence of spin-spin exchange interactions between these two hemes;
e) for cytbc_1 the number of species was 2, while the second species representing heme b_h was divided into two fractions with the lower and higher E_m and contributions of 60% and 40%, respectively.

The first and the second assumptions were supported by the observation, that increasing the number of species from 3 to 4 did not lead to any sensible results of the fit and the obtained parameters of the redundant 4th species (T_{dip}, fraction and E_m) were always very similar to one of the remaining species with shared contribution to compensate the respective fractions.
A model used for fitting to the whole experimental set of ESE decay curves is described by the following formula:

$$A(t,E_h) = A_{base}(t) \cdot (f_{base} + f_1(E_{m1}) \cdot exp(\frac{-t}{T_{dip1}}) + f_2(E_{m2}) \cdot exp(\frac{-t}{T_{dip2}}) + f_3(E_{m3}) \cdot exp(\frac{-t}{T_{dip3}}))$$

$$f_{base} + f_1 + f_2 + f_3 = 1$$

eq. S2

where: $A(t, E_h)$ is the amplitude of ESE decay curve at time point t [μs] of the ESE decay measured for a sample at particular E_h [mV]; $A_{base}(t)$ is the normalized experimental ESE decay curve (the base function) measured for the sample at the lowest E_h; f_{base} is the fraction of the base function at given E_h; f_1, f_2 and f_3 are the fractions of oxidized heme iron ions, that contribute to the enhancement

of the phase relaxation of 2Fe2S; T_{dip1}, T_{dip2} and T_{dip3} are the semi-quantitative time constants associated with the enhancement of the phase relaxation imposed on 2Fe2S by a respective fast relaxing species; t and E_h are independent variables representing the time axis of a single ESE decay curve and the external redox potential, respectively.

The fractions f_1, f_2 and f_3 (f_x) were calculated on the basis of the Nernst equation, as a function of E_m, n and E_h for a particular sample:

$$f_x = \frac{10^{0.01666 \cdot n \cdot (E_h - E_{mx})}}{1 + 10^{0.01666 \cdot n (E_h - E_{mx})}} \qquad \text{eq. S3}$$

where: f_x - fraction of species x, E_{mx} - the redox midpoint potential of species x. For the reasons given in the main text the parameter n was fixed at 1.

By combining eqs. S2 and S3, we obtained the model, in which all experimental ESE decay curves, at different E_h, were described by 9 global, free adjustable parameters: 3 E_m, 3 n and 3 T_{dip} values.

Measurement of E_h-dependent enhancement of the phase relaxation of 2Fe2S in cytb$_6$f. Global fitting of the model to ESE decay curves of cytb_6f with all 9 parameters to optimize was found to be unstable resulting mostly in unreliable values of *n,* which entails random combinations of E_m and T_{dip}. Fixing n values at 1 greatly improved the stability of the optimization. The convergence criteria was tested by performing 1000 independent optimizations, starting from randomized set of initial guesses for T_{dip}, E_m, and fixed n =1. Values of T_{dip} were randomly drawn from the range 0.1 to 20 μs, while E_m values were randomly drawn from the range -200 to +200 mV. Among 1000 optimizations 573 were successful, while 424 did not reached the global minimum, showing that a probability of successful optimization was 0.573 +/- 0.016. All successful optimizations yielded nearly the same values of T_{dip} and E_m for respective species. The results of the fitting was shown in Table 3 in the Main Text.

We also performed another fit with parameters of E_m and n fixed at values determined by optical and CW EPR spectroscopy (Figures 2 and 3): E_m = -111 mV and n=1 (species 1 - heme b_n); E_m = -80 mV and n = 0.7 (species 2 - heme b_p), E_m = +46 mV and n = 1, (species 3 - heme c_n). In this case, the only adjustable free parameters were T_{dip}.

In both these cases T_{dip} values are the lowest for the species 1 with the lowest E_m suggesting, that it is more remote from the 2Fe2S cluster than the species with intermediate E_m value.

Measurement of E_h-dependent enhancement of the phase relaxation of 2Fe2S in cytbc$_1$. We compared the results of measurement of ESE decay in cytb_6f to the data obtained in similar manner for cytbc_1. In this case the model was slightly modified to account for the presence of two fractions of heme b_h with higher (b_{hh}) and lower (b_{hl}) E_m. These two fractions of the heme b_h were expected to have the same dipolar effects on the phase relaxation of 2Fe2S as they correspond to the same structure, distance and paramagnetic properties. The fit with fixed E_m and n parameters (drawn from optical spectroscopy data) yielded T_{dip} of heme b_l less than that of heme b_h which could be expected as both these hemes possess similar paramagnetic properties and the difference in dipolar effect on phase relaxation of 2Fe2S results from heme b_l being closer to 2Fe2S than heme b_h.

The result of fitting of the above discussed models to ESE decay curves of cytb_6f and cytbc_1 are presented in Figure S4.

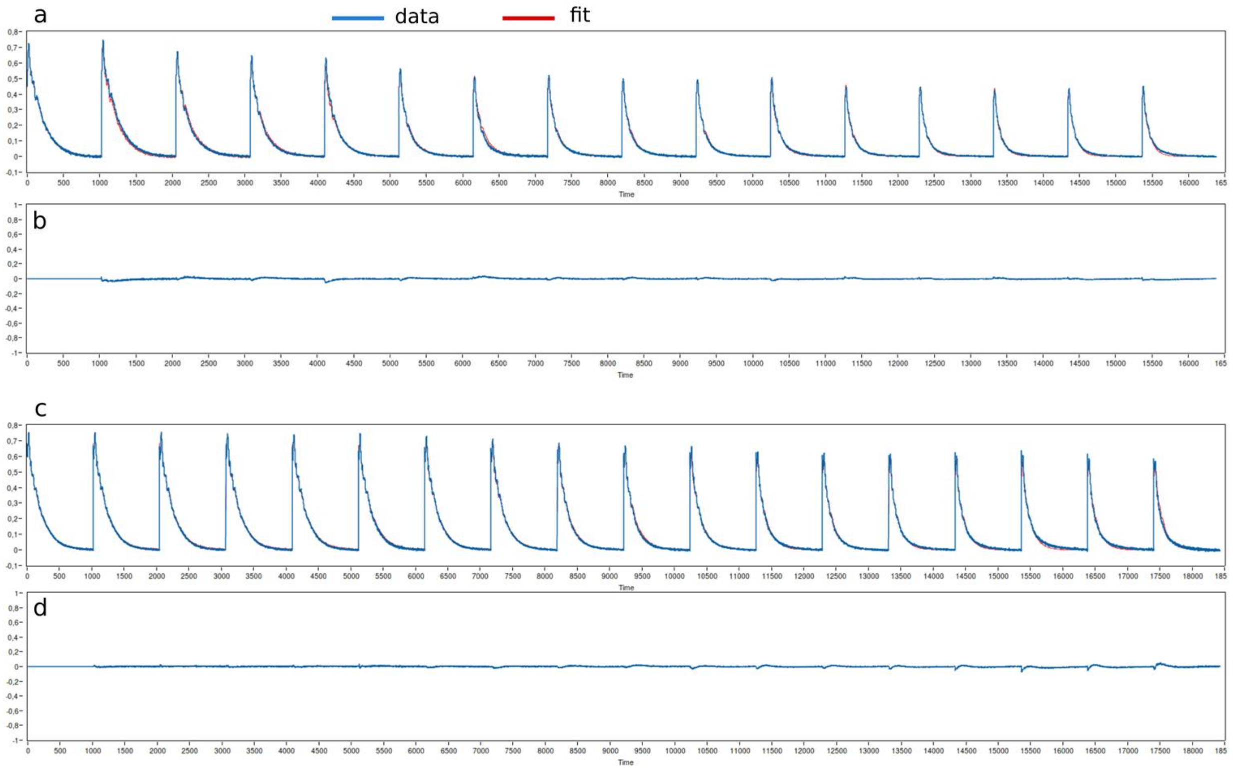

Figure S4. Results of application of GAF to ESE DC curves obtained for cyt*bc*$_1$ and cyt*b*$_6$*f*. a) the concatenated ESE DCs measured for cyt*b*$_6$*f* at different E_h (blue) and the GAF curves (red). b) Difference between ESE DCs and the fitted model. Panels c) and d) represent the same analysis as for a) and b), respectively but obtained for cytochome *bc*$_1$. The x-axes represent consecutive points of ESE DCs, that were concatenated starting from the lowest (left) to the highest E_h (right). Before analysis each ESE DC was normalized by division of ESE DC amplitude by estimated amplitude at the zero time point (the onset of the relaxation process). Initial 440 ns of the ESE DC are missing as being obscured by the resonator dead time.

Overall, the analysis of ESE decay curves measured for cyt*b*$_6$*f* provided estimates of E_ms for the 3 species but gave no explicit information on which heme was represented by the respective species. However, comparison of E_m values obtained by the analysis of relaxation curves with those obtained from optical and CW EPR spectroscopy (see Figures 2 and 3 in the Main Text) suggests that E_m of species 1 is similar to E_m of heme b_n (species having E_m around -129 mV (Figure 2b)) and therefore it is most probably heme b_n. Species 2, with E_m = - 96 mV has similar E_m value to heme b_p (Figure 2a). Both these comparisons correspond well with the spatial arrangement of hemes in the structure of cyt*b*$_6$*f* given that the T_{dip} obtained for species 1 is lower than that of species 2 and heme b_n is placed more remotely from 2Fe2S than heme b_p. Species 3 was optimized with E_m value around + 20 mV which is close to the value obtained for heme c_n (Figure 3b).

Discussion on relaxation enhancement

We performed analysis of pulse EPR data of cyt*b*$_6$*f* and the results were compared to the data obtained for structurally similar protein cyt*bc*$_1$. Figure 4 in the Main Text compares the distances between paramagnetic 2Fe2S center and heme iron ions in both cytochromes. In the case of cyt*bc*$_1$, heme b_l is positioned closer to 2Fe2S (approx. 26.4 Å) than heme b_h (approx. 34.2 Å), therefore assuming that these two hemes have similar paramagnetic properties the former heme should exert a stronger dipolar effect on the phase relaxation of the 2Fe2S cluster than the latter. Therefore, a shorter T_{dip} for heme b_l-2Fe2S interactions was expected. Similar effect was expected for cyt*b*$_6$*f* in which heme b_p is also closer to the 2Fe2S cluster than heme b_n (~ 30.5 Å and ~39.4 Å). Theoretically, dipolar relaxation curves are not mono-exponential as they depend on many additional factors such as relative angles between the vectors connecting the paramagnetic centers and the external magnetic field. However, we performed a qualitative comparison of the relative dipolar relaxation time constants by taking into account only relative distances and T_{dip} values of cyt*b*$_6$*f* and cyt*bc*$_1$. In the most simplified case, the strength of the dipolar effect on the phase relaxation can be described by a formula which considers distance *r* between two interacting centers:

$$\langle T_{dip}^{-1} \rangle = \langle a \rangle \cdot r^{-3} \qquad \text{eq. S4}$$

where: $<T_{dip}^{-1}>$ is the average, effective dipolar ESE decay rate caused by the dipolar interactions, $<a>$ is the proportionality factor including all remaining effective contributions other than distance averaged, while *r* is the distance between 2Fe2S cluster and respective heme iron ion. First we calculated average $<a>$ values for interactions between hemes b_p, b_n, c_n and 2Fe2S in cyt*b*$_6$*f* and corresponding $<a>$ for interactions between heme b_l and b_h and the 2Fe2S cluster in cyt*bc*$_1$ with the use of T_{dip} values obtained for the respective cytochromes. In case of cyt*b*$_6$*f*, the averaged $<a>$ value was estimated to ~27.5 Å^3×GHz, while for cyt*bc*$_1$ this value was similar and equal to ~30.0 Å^3×GHz. Using these different $<a>$ values for calculating distances between 2Fe2S cluster and hemes in both cytochromes and respective T_{dip} values, we obtained distances ~34.4 or ~36.8 Å for 2Fe2S - heme b_p, ~38.6 or ~39.4 Å for 2FeS2 - heme b_n and ~44.0 or ~41.7 Å for 2Fe2S - heme c_n. In cyt*bc*$_1$ the estimated distances were ~28.5 and ~32.1 Å for 2Fe2S - heme b_l and 2Fe2S - heme b_h, respectively. These values however must be treated only as crude approximations since such an analysis ignores many factors that contribute to the effective enhancement of the phase relaxation of the cluster. Most of the additional factors are not available and, additionally, we must take into account the fact that the PDB structures do not provide information on the distribution of the position of 2Fe2S due to the mobility of the iron-sulfur protein, an inherent feature of cyt*bc*$_1$ and cyt*b*$_6$*f*. However, despite the above-mentioned limitations in the analysis of the pulse EPR data, a general view drawn from these experiments is qualitatively in agreement with the expected larger effects of hemes b_p/b_l on the relaxation of 2Fe2S than hemes b_n/b_h due to the differences in their relative distances to the cluster and their estimated E_m values. This again supports the thesis that heme b_p in cyt*b*$_6$*f* exhibits a higher E_m than heme b_n, whereas in cyt*bc*$_1$ the respective heme b_l and b_h are low- and high potential LS heme.

References

(1) Baniulis, D.; Zhang, H.; Zakharova, T.; Hasan, S. S.; Cramer, W. A. Purification and Crystallization of the Cyanobacterial Cytochrome B6f Complex. *Methods Mol. Biol.* **2011**, *684*, 65–77. https://doi.org/10.1007/978-1-60761-925-3_7.

(2) Romanowska, E. Isolation of Cytochrome B6f Complex from Grana and Stroma Membranes from Spinach Chloroplasts. *Photosynth. Res. Protoc. Methods Mol. Biol. vol. 684* **2011**, *684*, 53–64. https://doi.org/10.1007/978-1-60761-925-3_6.

(3) Porra, R. J.; Thompson, W. A.; Kriedemann, P. E. Determination of Accurate Extinction Coefficients and Simultaneous Equations for Assaying Chlorophylls a and b Extracted with Four Different Solvents: Verification of the Concentration of Chlorophyll Standards by Atomic Absorption Spectroscopy. *BBA - Bioenerg.* **1989**, *975* (3), 384–394. https://doi.org/10.1016/S0005-2728(89)80347-0.

(4) Metzger, S. U.; Cramer, W. A.; Whitmarsh, J. Critical Analysis of the Extinction Coefficient of Chloroplasty Cytochrome F. *Biochim. Biophys. Acta - Bioenerg.* **1997**, *1319* (2–3), 233–241. https://doi.org/10.1016/S0005-2728(96)00164-8.

(5) Valkova-Valchanova, M. B.; Saribas, A. S.; Gibney, B. R.; Dutton, P. L.; Daldal, F. Isolation and Characterization of a Two-Subunit Cytochrome b-C1 Subcomplex from Rhodobacter Capsulatus and Reconstitution of Its Ubihydroquinone Oxidation (Q(o)) Site with Purified Fe-S Protein Subunit. *Biochemistry* **1998**, *37* (46), 16242–16251. https://doi.org/10.1021/bi981651z.

(6) Virtanen, P.; Gommers, R.; Oliphant, T. E.; Haberland, M.; Reddy, T.; Cournapeau, D.; Burovski, E.; Peterson, P.; Weckesser, W.; Bright, J.; van der Walt, S. J.; Brett, M.; Wilson, J.; Millman, K. J.; Mayorov, N.; Nelson, A. R. J.; Jones, E.; Kern, R.; Larson, E.; Carey, C. J.; Polat, İ.; Feng, Y.; Moore, E. W.; VanderPlas, J.; Laxalde, D.; Perktold, J.; Cimrman, R.; Henriksen, I.; Quintero, E. A.; Harris, C. R.; Archibald, A. M.; Ribeiro, A. H.; Pedregosa, F.; van Mulbregt, P.;

Vijaykumar, A.; Bardelli, A. Pietro; Rothberg, A.; Hilboll, A.; Kloeckner, A.; Scopatz, A.; Lee, A.; Rokem, A.; Woods, C. N.; Fulton, C.; Masson, C.; Häggström, C.; Fitzgerald, C.; Nicholson, D. A.; Hagen, D. R.; Pasechnik, D. V.; Olivetti, E.; Martin, E.; Wieser, E.; Silva, F.; Lenders, F.; Wilhelm, F.; Young, G.; Price, G. A.; Ingold, G. L.; Allen, G. E.; Lee, G. R.; Audren, H.; Probst, I.; Dietrich, J. P.; Silterra, J.; Webber, J. T.; Slavič, J.; Nothman, J.; Buchner, J.; Kulick, J.; Schönberger, J. L.; de Miranda Cardoso, J. V.; Reimer, J.; Harrington, J.; Rodríguez, J. L. C.; Nunez-Iglesias, J.; Kuczynski, J.; Tritz, K.; Thoma, M.; Newville, M.; Kümmerer, M.; Bolingbroke, M.; Tartre, M.; Pak, M.; Smith, N. J.; Nowaczyk, N.; Shebanov, N.; Pavlyk, O.; Brodtkorb, P. A.; Lee, P.; McGibbon, R. T.; Feldbauer, R.; Lewis, S.; Tygier, S.; Sievert, S.; Vigna, S.; Peterson, S.; More, S.; Pudlik, T.; Oshima, T.; Pingel, T. J.; Robitaille, T. P.; Spura, T.; Jones, T. R.; Cera, T.; Leslie, T.; Zito, T.; Krauss, T.; Upadhyay, U.; Halchenko, Y. O.; Vázquez-Baeza, Y. SciPy 1.0: Fundamental Algorithms for Scientific Computing in Python. *Nat. Methods* **2020**, *17* (3), 261–272. https://doi.org/10.1038/s41592-019-0686-2.

(7) Harris, C. R.; Millman, K. J.; van der Walt, S. J.; Gommers, R.; Virtanen, P.; Cournapeau, D.; Wieser, E.; Taylor, J.; Berg, S.; Smith, N. J.; Kern, R.; Picus, M.; Hoyer, S.; van Kerkwijk, M. H.; Brett, M.; Haldane, A.; del Río, J. F.; Wiebe, M.; Peterson, P.; Gérard-Marchant, P.; Sheppard, K.; Reddy, T.; Weckesser, W.; Abbasi, H.; Gohlke, C.; Oliphant, T. E. Array Programming with NumPy. *Nature* **2020**, *585* (7825), 357–362. https://doi.org/10.1038/s41586-020-2649-2.

(8) McKinney, W. Data Structures for Statistical Computing in Python. *Proc. 9th Python Sci. Conf.* **2010**, *1* (Scipy), 56–61. https://doi.org/10.25080/majora-92bf1922-00a.

Author Contributions

MSz isolated and purified proteins. MSz, ŁB and MS designed and performed experiments and analyzed data. AO acquired funding and discussed data, All authors participated in writing the manuscript.
